# The Importance of Redox Status in the Frame of Lifestyle Approaches and the Genetics of the Lung Innate Immune Molecules, SP-A1 and SP-A2, on Differential Outcomes of COVID-19 Infection

**DOI:** 10.3390/antiox9090784

**Published:** 2020-08-25

**Authors:** Fotios Tekos, Zoi Skaperda, Nikolaos Goutzourelas, David S. Phelps, Joanna Floros, Demetrios Kouretas

**Affiliations:** 1 Department of Biochemistry-Biotechnology, University of Thessaly, 41500 Larissa, Greece; fotis.tekos@gmail.com (F.T.); zoiskap94@gmail.com (Z.S.); nikgkoutz@gmail.com (N.G.); 2 Center for Host Defense, Inflammation, and Lung Disease (CHILD) and Departments of Pediatrics, Hershey, PA 17033, USA; dsp4@psu.edu (D.S.P.); jfloros@pennstatehealth.psu.edu (J.F.); 3 Obstetrics & Gynecology, Pennsylvania State University College of Medicine, Hershey, PA 17033, USA

**Keywords:** COVID-19, SARS–Cov-2, free radicals, oxidative stress, antioxidants, exercise, fasting, surfactant protein A

## Abstract

The pandemic of COVID-19 is of great concern to the scientific community. This mainly affects the elderly and people with underlying diseases. People with obesity are more likely to experience unpleasant disease symptoms and increased mortality. The severe oxidative environment that occurs in obesity due to chronic inflammation permits viral activation of further inflammation leading to severe lung disease. Lifestyle affects the levels of inflammation and oxidative stress. It has been shown that a careful diet rich in antioxidants, regular exercise, and fasting regimens, each and/or together, can reduce the levels of inflammation and oxidative stress and strengthen the immune system as they lead to weight loss and activate cellular antioxidant mechanisms and reduce oxidative damage. Thus, a lifestyle change based on the three pillars: antioxidants, exercise, and fasting could act as a proactive preventative measure against the adverse effects of COVID-19 by maintaining redox balance and well-functioning immunity. Moreover, because of the observed diversity in the expression of COVID-19 inflammation, the role of genetics of innate immune molecules, surfactant protein A (SP-A)1 and SP-A2, and their differential impact on the local lung microenvironment and host defense is reviewed as genetics may play a major role in the diverse expression of the disease.

## 1. Introduction

**Figure 1:**
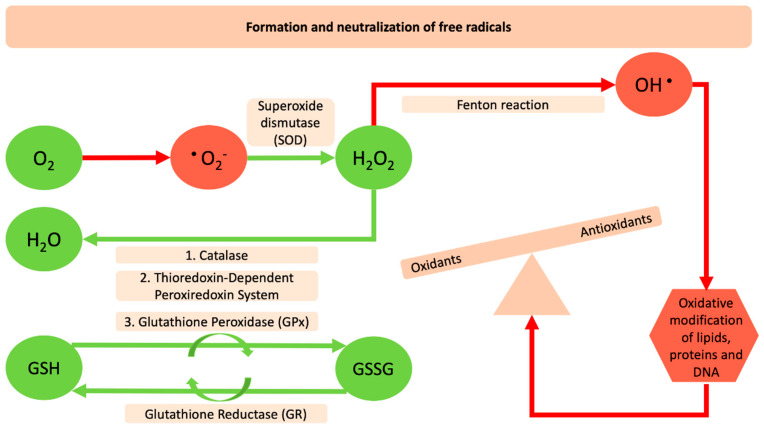
Formation and neutralization of free radicals. The red arrows and red shapes indicate the harmful reactive oxygen species and their negative impact on the oxidation of macromolecules. The green arrows and shapes indicate the antioxidant enzymes and the reactions that counteract and neutralize oxidants.

December of 2019 may represent an era that will change the mindset of humanity from its roots. The “severe acute respiratory syndrome coronavirus-2 (SARS-CoV-2)”, provoked a highly contagious disease that was first reported in Wuhan city in China and within months spread throughout the globe. This posed the greatest challenge we have faced since World War Two, and was declared a global pandemic by the World Health Organization (WHO) on 11th of March, 2020 [1]. By the end of May 2020, the virus had infected more than 6 million people worldwide and killed more than 370,000. Inflammation, cell death, and other pathophysiological processes have been extensively linked with respiratory viral infections that could be linked also with a redox imbalance or oxidative stress. Reactive Oxygen Species (ROS) are molecular species with well-established physical–chemical properties [2]. Four successive steps of one-electron reduction starting with molecular oxygen are necessary for their production (Figure 1). There are three major species, the superoxide anion (O_2_^•−^), hydrogen peroxide (H_2_O_2_) and the hydroxyl radical (HO^•^) [3]. O_2_^•−^, and HO^•^ can participate in reactions with organic substrates and produce intermediate species that can lead to further formation of other ROS. ROS can be detrimental for DNA, proteins and lipids because of their ability to induce oxidative modifications of these molecules that are involved in several pathologic conditions [4]. The human body has endogenous antioxidant defense systems that eliminate reactive species such as superoxide anions (O_2_^•‒^), hydrogen peroxide (H_2_O_2_), and the hydroxyl radical (HO^•^). Enzymatic antioxidants such as, catalases (CAT) superoxide dismutase (SOD), the thioredoxin-dependent peroxiredoxin system, glutathione peroxidases (GPxs) together with the non-enzymatic antioxidants can counteract free radicals and neutralize oxidants [5]. The specific role of SOD is to catalyze the dismutation of superoxide radicals (O_2_^−^) to molecular oxygen (O_2_) and hydrogen peroxide. The hydrogen peroxide molecule is reduced further to water using glutathione peroxidase (GPx), which in the next step is recharged by glutathione reductase (GR) and this, in turn, is recharged by a number of antioxidant compounds. Furthermore, CAT plays a role in the conversion of hydrogen peroxide into oxygen and water [6] (Figure 1). This antioxidant defense mechanism can be deregulated by several pathologic conditions such as diabetes, hypertension, and obesity. The deregulation of the redox state and the levels of molecules and the related enzyme activities and the crosslink with several diseases seems to be one of the main concerns that many toxicological fields have tried to describe for decades.

Moreover, the lung is constantly exposed to external environment threats, such as bacteria, viruses, toxins, allergens, and other that have the potential to increase inflammation and redox imbalance. However, lung innate immune cells (i.e., the alveolar macrophage) and innate immune molecules such as the surfactant proteins, (SP-A)1 and SP-A2, as well as others, by providing the first line of host defense against the various insults, maintaining lung health under normal circumstances. SP-A1 and SP-A2 are components of pulmonary surfactant, a lipoprotein complex, the key function of which is to reduce surface tension in the distal lung (i.e., alveoli) and prevent lung collapse, and thus enable the O_2_/CO_2_ exchange to occur in the lung, an essential function for life [7]. In fact, SP-A1 and SP-A2 variants have been shown previously to differentially regulate inflammatory processes and host defense [8,9,10], and mice, each with a different SP-A variant showed significantly different survival rates [11] and lung function [12] after *K. pneumoniae* infection. Collectively, the surfactant components play important roles in surfactant-related functions and in the regulation of inflammation and innate host defense, and the surfactant proteins, in particular, are involved collectively in all these functions/processes. In most, if not all, pulmonary diseases there is surfactant dysfunction and/or dysregulation of inflammatory processes and host defense, and in this context optimally functioning surfactant components are central to nearly all pulmonary diseases.

The issues addressed in this review are several-fold. First, we will review whether the redox-related mechanisms are deregulated in the COVID-19 infection. Second, we will investigate lifestyle changes that have been shown to positively affect redox balance whether these could, if applied, position individuals in a more advantageous situation for subsequent waves of COVID-19 infection. Third, we will review whether the genetics of innate immune molecules, SP-A1 and SP-A2, that have been shown previously to differentially affect the alveolar microenvironment, play a role in determining the severity of COVID-19 infection and thus explain, in part, the variable clinical outcome in these patients. Moreover if the answer is yes, what kind of therapeutic interventions may be considered?

## 2. The Interrelation between Reactive Oxygen Species and SARS-Cov-2

**Figure 2:**
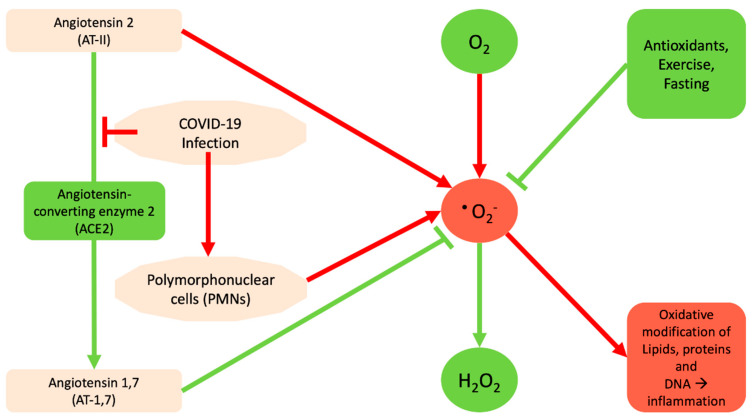
Diagram indicating how COVID-19 may contribute to the generation of free radicals. The red arrow and red shapes indicate processes as well as the contributions of COVID-19 to the generation of reactive oxygen species (ROS). The negative impact of ROS on the macromolecules and on subsequent inflammatory processes is shown. Green arrows/line and green shapes indicate processes and/or enzymes that eliminate ROS or prevent its generation.

Based on analysis of decade-long structural studies of SARS Coronavirus, researchers have confirmed the binding of SARS-CoV to the angiotensin-converting enzyme 2 (ACE2) receptor [13]. Thus it appears that the new coronavirus binds optimally to the human receptor ACE2 [14,15,16]. The major role of ACE2 is to lower the blood pressure by catalyzing the hydrolysis of angiotensin 2 (AT-II) into AT-1,7 [17,18,19]. AT-1,7 inhibits the pro-oxidant effects of AT-II, through reduction of superoxide anion production, while AT-II promotes it [20]. Therefore, basically the binding of SARS-CoV-2 to ACE2 is going to inactivate it and prevent the production of AT-1,7, and consequently deregulate superoxide levels. Moreover, the COVID-19-mediated inflammation is likely to increase the amount of AT-II that subsequently increases the amount of superoxide (Figure 2). Regarding the potential protective effects of AT-1,7 the existing data indicate that treatments based on ACE2 approaches might be promising strategies in order to minimize aberrant vascular responses and atherothrombosis. Specifically, ACE2, by means of the AT-1,7 system, exerts its role based on a mechanism that might involve attenuation of Nicotinamide Adenine Dinucleotide Phosphate (NADPH)-oxidase-induced ROS production, thus leading to the improvement of endothelial homeostasis [21]. Furthermore, ACE2 and AT-1,7 significantly prevent early atherosclerotic lesion formation by means of protection of endothelial function, counter-regulation of AT-II signaling and inhibition of inflammatory response [22]. Likewise, recently it has been shown that in women with preeclampsia, AT-1,7 showed increased endothelium-dependent vasodilation via nitric oxide (NO) synthase-mediated pathways and also attenuated AT-II–mediated constriction. This indicates that women with preeclamptic pregnancy may have a chance for a viable therapeutic target, the AT-1,7 [23].

Another problem resulting from COVID-19 is that it causes inflammation and specifically it recruits neutrophils or polymorphonuclear cells (PMNs) [24] that are able to use NADPH oxidase to stimulate the conversion of oxygen to superoxide [25]. The connection of NADPH oxidase, AT-II, and the way that it can cause oxidative stress comes from a study regarding atherosclerosis. In that study NADPH oxidase and O_2_^−^ production are increased in vascular cells by a variety of agonists relevant to the pathogenesis of atherosclerosis, including AT-II [26]. Subsequent to this, it was shown that there are several enzyme systems contributing to the formation of ROS, including NADPH oxidase and equally how AT-II stimulates, both in vitro and in vivo, increases in ROS [27]. With respect to the PMNs and the innate immune state of SARS-Cov-2 infected patients, there is still limited information. Specifically, one study with 99 cases from Wuhan found increased total neutrophils (38%), reduced total lymphocytes (35%), increased serum interleukin 6 (IL-6) (52%) and increased c-reactive protein (C-RP) (84%). Moreover, another study showed statistically different increases in total neutrophils and decreases in total lymphocytes in severe patients of ICUs compared with those in non-ICU care [28]. Neutrophils, on the other hand, may activate the production of ROS by NADPH oxidases [29].

Given the limited information with regards to the characteristics and outcomes of the new coronavirus patients that develop severe symptoms, there is an urgent need to describe the specifics of other underlying diseases these patients may have. Among 5700 patients hospitalized with COVID-19 infection in the New York City area the number of patients that were hospitalized and had hypertension were 57% of the total 5700 patients versus 7% in the general population. In addition patients who had BMI >30% were 42% of the total 5700 patients that were admitted to the hospital versus 30% in the general population [30]. Moreover 11% of the 5700 patients had coronary artery disease and 7% had congestive heart failure [30]. These numbers are higher than they are in the general community. Moreover, those who were admitted with asthma were 9%, with chronic obstructive pulmonary disease (COPD) 5%, and obstructive sleep apnea 3%, and these percentages are about the same as they are in the general population [30], indicating that these conditions may not be a really big risk factor. On the other hand, it seems that COVID-19 affects people with cardiovascular disease and obesity. A common thing between these two pathological conditions is the level of oxidative stress. It is well-known that obese, cardiovascular patients, and aged people have deregulated redox states and definitely tip the balance in favor of oxidative stress [31].

**Figure 3:**
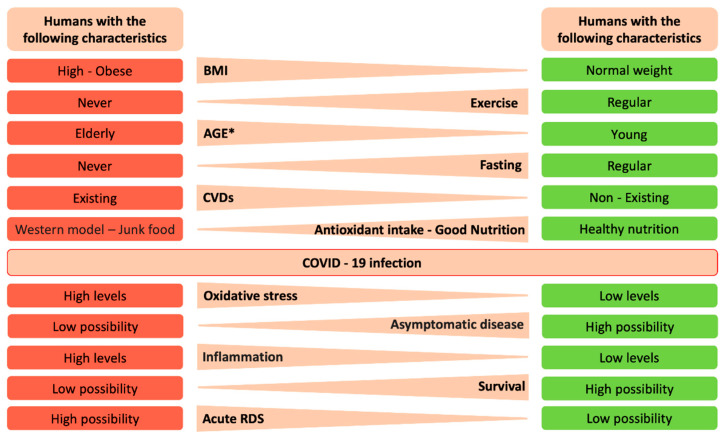
Human characteristics and potential outcomes after infection with severe acute respiratory syndrome coronavirus-2 (SARS-CoV-2). In this figure we summarize lifestyle characteristics and the underlying mechanisms as well as potential outcomes before or after SARS-CoV-2 infection. * In the present context age, young and old, means young or old in chronological age, but also could refer to young and old in terms of their lung health (i.e., a chronologically old person may have “young” physiological indices due to healthy lifestyle habits, and vice versa).

A critical aspect of these conditions is that oxidative stress leads to cardiovascular diseases. It is known that early events in cardiovascular disease (CVD) occur when the vascular endothelium is damaged. This damage impairs the function of the endothelium, a condition called endothelial dysfunction [32]. The physiological function of endothelium can be modified directly by activating several transcription factors leading to the upregulation of adhesion molecules in platelets and leukocytes and decreasing the bioavailability of nitric oxide (NO) or indirectly by increasing the formation of advanced glycation end products (AGEs) or increasing oxidation of low density lipoproteins [33]. Because of the fact that the endothelium represents the main regulator of the vascular wall, homeostasis preserves low levels of oxidative stress both by releasing mediators such as NO and by controlling local AT-II activity [34]. Therefore, endothelial dysfunction is associated with decreased synthesis of NO from endothelial NO synthase and increased oxidative inactivation of NO [35]. SOD, CAT, GPx, and glucose-6-phosphate dehydrogenase deficiency G6PD are critical antioxidant enzymes that eliminate ROS and can inactivate NO. Deficiencies of the aforementioned enzymes lead to increased oxidative stress and NO inactivation and as such can contribute either directly or indirectly through other mechanisms to Endothelial Cell Dysfunction (ECD) [36]. To prevent the dysfunction of the antioxidant defense system selected antioxidants have been used for a long time in an attempt to improve the intracellular redox state and prevent pathological conditions that we described above [37]. Until now we have explored evidence that provide connection between CVDs, aging, and high BMI in humans with redox imbalances and potentially with COVID-19 infection. Below we will discuss further specific endpoints as shown in Figure 3 that may differentially be expressed in individuals that express severe COVID-19 infection and those who do not.

## 3. Lifestyle Changes

Lifestyle changes such as systematic and chronic exercise, diet, and fasting are shown to improve physiological fitness and health, in general. As the future with COVID-19 is currently uncertain with the possibility of a second or more waves of the disease occurring in the coming year(s), it is prudent to think ahead and consider ways as to how one may best position himself/herself to better fight or attenuate the severity of the disease or even prevent it altogether. Below we discuss lifestyle changes, such as natural antioxidant regimens, exercise, and fasting, among others, to promote antioxidant mechanisms and reduce inflammation.

### 3.1. Antioxidants and Natural Dietary Regimens that Promote a Strong Host Antioxidant Environment

The redox-related mechanism of the infectious action of SARS-CoV-2, i.e., the induced lung toxicity via the enhancement of reactive oxygen species generation could provide a clue regarding the use of specific compounds with antioxidant potency against its virulence [38] and the fortification of the antioxidant arsenal of the host. An enhanced antioxidant status of the host cells may be a significant factor against infection, given that there are many examples of viruses and pathogens in general that are eliminated in an antioxidant environment [39]. An interesting proposal is to use N-acetylcysteine (NAC), which is a precursor molecule for reduced Glutathione (GSH) as a therapeutic intervention [38]. The infection by SARS-CoV-2 is thought to induce accumulation of viral and other proteins that are not degraded by proteasome and, hence, cause toxic effects in the infected cell by disrupting redox homeostasis [40]. Therefore, NAC administration could be considered a reasonable approach since NAC inhibits proteasome inhibitors [40]. In addition to NAC, whey protein administration seems to be another promising idea for the enhancement of the host antioxidant status and for increasing possibilities for the attenuation or prevention of COVID-19. Whey protein is a by-product of the cheese making process in dairy industry and due to its high organic burden it is considered a serious pollutant for the environment when discarded without control [41]. Nevertheless, recent studies have shown that it is a very potent antioxidant agent of animal origin and its beneficial properties are similar to the traditionally established extracts of plant origin [42,43]. Interestingly, sheep/goat whey protein has been extensively examined for its beneficial action in several experimental environments, namely in cell lines and in vivo, with promising results [44]. Indeed, whey protein is shown to be a strong antioxidant and antimutagenic agent in cell culture [45,46,47] and in vivo [48,49,50], and it appears to protect against inflammation, a low-grade inflammatory state that makes the elderly vulnerable to COVID-19 [38,51]. Under an appropriate administration regimen, whey protein could fortify the antioxidant defense of the host and act as an important preventive agent against SARS-CoV-2 virulence given that several parameters such as the route of administration, the nutritional and health status of the individuals, the personalized regimen for antioxidant administration, and others will be taken into account.

In addition to the above, another class of antioxidant molecule, polyphenols, are of particular interest. Polyphenols are found mainly in plant foods such as fruits and vegetables, but also in their derivatives, such as wine and olive oil. Several studies have shown that many foods rich in or enriched with polyphenols can have a positive effect on oxidative stress indicators in in vitro and in vivo studies. Studies in the EA.hy926 cell line have confirmed the antioxidant activity of polyphenolic grape and olive extracts by improving GSH levels [52,53,54,55]. Additionally, studies in livestock such as pigs, hens, and sheep, have found that animal feed supplemented with antioxidants derived from olive oil mill wastes and wine bioproducts, improved the redox status in animals [56,57,58,59]. Finally, a study in humans that consumed a significant amount of pomegranate juice resulted in increased GSH levels and reduced lipid peroxidation [60]. The molecular mechanisms by which polyphenols exert their antioxidant activity are connected with nuclear factor erythroid 2–related factor 2 (NRF2) [61,62,63]. Activating the NRF2–Kelch-like ECH-associated protein 1 (KEAP1) pathway leads to the activation of the expression of genes controlled by the antioxidant response element (ARE). ARE controls a number of genes with antioxidant, detoxifying and anti-inflammatory action [64]. Polyphenols can therefore strengthen the antioxidant defense, and their intake through diet could be a potential protection mechanism against SARS-CoV-2.

### 3.2. Exercise

Scientists are constantly discovering new benefits of exercise. In experiments over the past 10 years, a number of studies have found that exercise can help with respiratory problems [65]. It has been shown that exercise promotes the activation of the body’s antioxidant mechanisms. Aerobic endurance exercise has been shown to activate the production of an antioxidant enzyme peroxide dismutase (EcSOD) that breaks down the superoxide root in healthy and obese people or people with type 2 diabetes [66,67,68,69,70]. SOD is the only antioxidant enzyme that breaks down the root of O_2_ to H_2_O_2_ and thus deactivates it. In addition, regular aerobic exercise can increase levels of the most important antioxidant molecules, GSH and GPx, where the latter uses GSH to neutralize free radicals [69,71] (Figure 2). Of particular interest, however, is the fact that several studies have shown that long-term aerobic exercise reduces oxidative damage in biomolecules. Several studies have shown that various aerobic exercise protocols that lasted at least several weeks led to a reduction in lipid peroxidation as seen in the reduction of thiobarbituric acid reactive substances (TBARS) and malondialdehyde (MDA) [66,69,71,72,73,74,75]. Whether a systematic long-term exercise routine could reduce the risk of death from COVID-19 remains to be determined. However, based on the available scientific data we postulate that a well-conditioned, fit individual, is highly likely to enjoy a strong antioxidant environment where pathogens, such as SARS-CoV-2, will not fare well and the risk for severe disease could be minimized.

### 3.3. Fasting

Humans fast daily during their sleep, which typically equals to 8–10 h. There are, however, periods of fasting that are imperatively longer than the periods of normal food intake. For example, fasting was a quite frequent situation in prehistoric times because access to food was difficult and as a result individuals of that era were obliged to survive without food until it was available again [76]. Nowadays, humans fast mainly for religious or health reasons [77,78,79,80,81]. To this end, believers abstain from food intake during certain periods dictated by their religion, such as Lent for Christians and Ramadan for Muslims, while patients go through a short fasting period before an operation or in their effort to improve the quality of their life by losing weight and body fat [82]. On the basis of the recently obtained scientific evidence, though, the residents of the so-called developed world follow fasting protocols without any obvious health reasons but just for taking advantage of the beneficial properties it offers to their psychological equilibrium and metabolic health [80].

Fasting is divided into two major categories, namely intermittent and periodic (or prolonged) fasting. Intermittent fasting, which is the most widespread, involves alternations between periods of fasting and food intake on a daily basis [83,84]. Usually the fasting period is 14–20 h and the feeding window lasts 10–4 h. Intermittent fasting has been extensively studied during the last decade in healthy individuals as well as in patients that mainly suffer from diabetes, metabolic syndrome, and obesity [83,84,85]. The available literature shows that intermittent fasting improves human health in terms of not only weight and fat loss but also of changes in insulin and growth hormone [76]. Improvements in biochemical indices, such as glucose, triglycerides, and cholesterol have also been reported [86]. On the other hand, periodic fasting refers to total abstinence from food or a restricted number of calories for 24 h to a few days. There is limited evidence on animals to indicate whether periodic fasting has beneficial for one’s health. Penguins and elephant seals are animals with the distinguishing characteristic that they normally fast for prolonged time periods. Relevant studies have demonstrated that Nrf2 is activated, thus protecting against oxidative damage through strengthening the antioxidant mechanism of these animals [87,88]. A recent study of long-term fasting models applied in humans showed improved blood redox status after a period of 10 days [89].

Among the beneficial effects of fasting, it has been observed that fasting decreases the levels of mammalian target of rapamycin (mTOR), which is associated with reduced cell growth and nuclear factor-κB (NF-κB) and this is, in turn, is correlated with reduced inflammation [90,91].

Furthermore, fasting results in the enhancement of AMP-activated protein kinase (AMPK) and Sirtuin (SIRT) levels that play a pivotal role in mitochondrial biogenesis, Forkhead box O (FOXO) that activates cellular autophagy, and Nrf2 that activates genes associated with antioxidant activity [92,93,94]. The beneficial effects of fasting interventions on several readouts, have been shown in numerous studies. These readouts include weight loss, metabolic shift from carbohydrate to fat burning, alteration in key hormone levels (e.g., adiponectin and leptin etc.), as well as improvements in inflammatory biomarker levels, such as brain-derived neurotrophic factor (BDNF), tumor necrosis factor alpha (TNF-α), interleukins, and C-RP [95,96,97,98,99,100,101,102]. In aggregate the collective published data indicate that fasting plays a significantly beneficial role, and pathological conditions such as obesity and cardiovascular diseases can be significantly altered by changes in food intake. This, in turn, may help these and other patients if infected with COVID-19 to perhaps experience reduced severity and avoid death.

## 4. Genetic Complexity of the Lung Innate Immune Molecules, SP-A1 and SP-A2, and Its Role in Lung Health

As noted above, surfactant protein-A (SP-A), is an innate immune molecule that plays important roles both in surfactant-related functions and innate immunity of the lung. In this section, we will briefly review the role of SP-A genetics on the regulation of the alveolar macrophage, alveolar microenvironment, survival outcomes, and pulmonary disease susceptibility, and discuss how this knowledge may apply to COVID-19 and/or how SP-A variants, by providing the first line of defense, may contribute to COVID-19 outcomes.

### 4.1. What have We Learned from Preclinical and/or Human Studies with Regards to the Role of SP-A Genetics on Lung Health?

**Figure 4:**
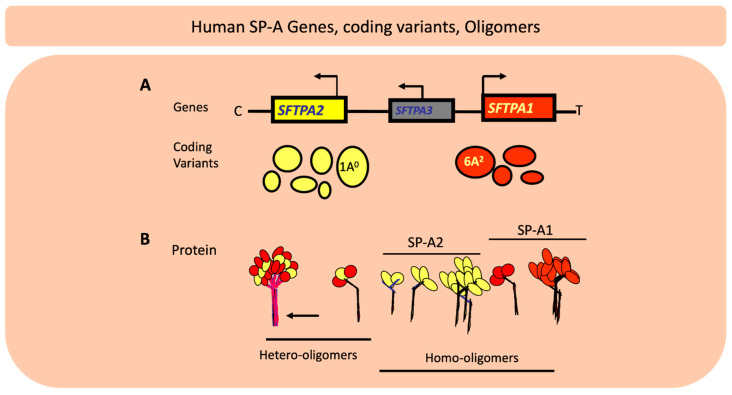
The chromosomal organization of *SPFTPA* locus consists of two functional genes the *SFTPA2* encoding SP-A2 and the *SFTPA1* encoding surfactant protein A (SP-A1) as well as a pseudogene, the *SFTPA3* as shown in (**A**). The location of the centromere (C) and the telomere (T) are depicted. The transcriptional orientation is shown by a bent arrow. The two functional genes have opposite transcriptional orientation. The coding variants for each gene are shown below the relevant gene and the size of the circle denotes the frequency of each in the general population with the 1A^0^ and 6A^2^ being the most frequently observed variants. (**B**) depicts hetero-oligomers and homo-oligomers.

**Figure 5:**
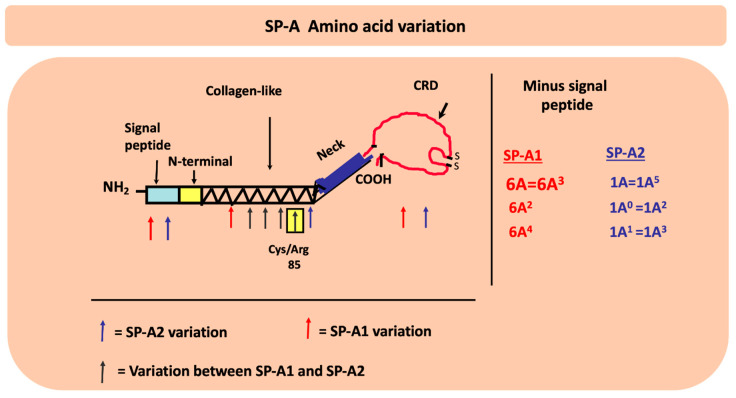
The location of the amino acid differences between the SP-A1 and SP-A2 genetic variants as well as the amino acid differences among variants are shown on the different SP-A protein regions (signal peptide, N-terminal, collagen-like, neck, and carbohydrate recognition domain (CRD)). The black arrows show the gene specific differences where all the SP-A1 variants differ from all the SP-A2 variants. The blue and red arrows show differences in SP-A2 and SP-A1 variants, respectively. Residue 85 is noted for its importance in function and biochemical properties between SP-A1 and SP-A2. After the signal peptide is removed, as shown, some of the variants become identical in their coding region.

In humans SP-A exhibits extensive genetic and epigenetic complexity [103]. Two genes, *SFTPA1* and *SFTPA2*, on chromosome 10 [104,105], encode SP-A1 and SP-A2, respectively, and each has been identified, in addition to splice and other sequence variants in their untranslated regions, with several coding variants [106,107,108]. Figure 4 Panel A depicts the chromosomal organization of the SP-A locus and the frequency of the SP-A1 and SP-A2 coding variants. The SP-A2 1A^0^ and the SP-A1 6A^2^ variants are the most frequently found in the general population, as depicted here with the largest circles. The other variants shown by smaller size circles indicate a lower relative frequency. For the SP-A1 variants the frequency sequence in the general population is 6A^2^>6A^3^>6A^4^>6A and for the SP-A2, 1A^0^>1A^1^>1A>1A^2^>1A^3^>1A^5^ [109]. SP-A1 and SP-A2 are found (Panel B) as hetero-oligomers [110] but homo-oligomers have been shown to be functional as well [111]. The SP-A molecule (Figure 5) consists of a number of regions including the signal peptide, N-terminal, collagen-like region, neck, and the carbohydrate recognition domain (CRD). SP-A1 and SP-A2 variants are distinguished between themselves by four amino acids within the collagen region and the SP-A1 and SP-A2 variants in addition to these four gene-specific differences differ in amino acids located in other regions of the protein, as shown in Figure 5, except for the neck region that is conserved among all known variants [107,112]. Residue 85 is a Cys in SP-A1 and an Arg in SP-A2 and plays a key role in function and biochemical properties of SP-A variants [113]. Residue numbering is based on the precursor molecule that includes the signal peptide. A number of the variants, as shown in Figure 5, are identical in amino acid sequence after the signal peptide is removed and thus these are expected to be similar in function although nothing is known about their regulation and hence their levels may differ. In addition to coding variants there are splice variants at the 5′ UTR [107,114] as well sequence variants at the 3′UTR [115,116]. The coding variants hold the potential for functional differences among themselves. In fact, a considerable body of research has shown this to be the case in both host defense and surfactant-related functions [8,9,10,11,12,117,118,119,120,121,122,123,124,125,126]. Although in terms of host defense, the mechanistic details of SP-A1/SP-A2 binding to alveolar macrophages to bring about the observed differential outcomes are not known, the B_max_ of SP-A2 binding to alveolar macrophage is 2–3 times higher than that of SP-A1 [127]. This is consistent with the higher activity the alveolar macrophages exhibit in response to SP-A2 as noted below, in terms of bacterial phagocytosis, cytokine production, among others. Moreover, a number of studies have shown associations of the surfactant protein variants with many pulmonary diseases further supporting a contribution of the surfactant proteins to pulmonary disease susceptibility and/or severity [128,129]. The association of the surfactant protein variants with nearly all the pulmonary diseases studied is not surprising because virtually in all pulmonary diseases there is a component of dysregulation of host defense/inflammatory processes and/or surfactant dysfunction or deficiency and the surfactant proteins play a role in both of these groups of functions, as noted above.

SP-A1 and SP-A2 variants differentially affect alveolar macrophage function (i.e., its ability to produce proinflammatory cytokines and carry out bacterial phagocytosis) [8,9,10,130,131]. Mouse models, where each mouse line carries and expresses a different human SP-A1 or SP-A2 variant or both, showed a differential impact on the bronchoalveolar lavage proteome [118], as well as the alveolar macrophage proteome [121,124], miRNome [117,119], and gene expression [132] under baseline conditions and in response to ozone-induced oxidative stress (OxS) or infection, and these responses were sex-specific. Furthermore, SP-A1 and SP-A2 variants have also been shown to differentially affect the epithelial type II cell miRNome in a sex-specific manner [120]. All these SP-A variant-dependent differential effects in the alveolus and alveolar cells indicate that the alveolar (local) microenvironment differs among SP-A variants and it is likely that these differences in the face of an insult combine to differentially affect downstream effects dictating the individual’s health and survival.

Preclinical animal studies have shown that both lung function [12] and survival [11] after bacterial infection varies significantly according to SP-A genotype, and sex. An SP-A genotype-dependent survival was also observed in humans after lung transplantation [133]. In these patients, survival was significantly more favorable, especially the first year after lung transplantation, which is the most critical time for these patients, if the donor lung carried a specific SP-A2 (1A^0^) genotype. Previous studies had shown that low levels of SP-A were associated with poor clinical outcome and early lung transplant survival [134]. Moreover, SP-A variants differentially affect surfactant-related processes [122,123,125], which, in turn, may compromise under certain circumstances the key function of the lung, the O_2_/CO_2_ exchange and ultimately the survival of the individual.

The animal and human studies underscore the importance of innate immunity and specifically of the SP-A variants in survival in response to various insults. Furthermore, the genetic associations between SP-A variants, and disease susceptibility and/or severity in a large number of pulmonary diseases [122,123,125] provide strong evidence of its role in lung health and disease. From the collective literature, it is clear that the SP-A genetic variability translates into functional diversity, and thus dysregulation and/or dysfunction of SP-A variants can affect surfactant function, host defense, regulation of inflammation, and other.

In summary, the available data together indicate: (a) the importance of SP-A in lung health; (b) that the differential effect of SP-A genetic variants on the proteome of the bronchoalveolar lavage and on the alveolar cells (function and regulation) may create a variable alveolar microenvironment that is SP-A genotype-dependent; (c) that the SP-A variant-dependent changes in the collective microenvironment (cellular and the surrounding hypophase/liquid) are sex-specific [11,12,117,118,119,120,121,126] and SP-A-concentration dependent [124]; (d) in a large number of pulmonary diseases, susceptibility and severity have been associated with SP-A genetic variants [122,123,125]. Moreover, although it is known that SP-A1 and SP-A2 are differentially regulated [114,135,136,137,138,139,140,141], it is currently unknown how this regulation may be further altered in the presence, for example, of infection. We postulate that the range of the variant-specific, sex-specific, and SP-A concentration-dependent differences under normal (unprovoked) healthy conditions do not have any significant negative impact on lung health but in the face of varied insults (bacteria, virus, OxS, allergens, other) these differences may be magnified, nullified, or altered to exhibit synergistic or additive effects, and as a result play a key role in determining susceptibility (risk or protection) and/or severity of the disease. This is supported, in part, by the observation that in humans with no known pulmonary disease, although the ratio of SP-A1 to total SP-A in the bronchoalveolar lavage varies [142], this ratio increases significantly in those with asthma, cystic fibrosis, and bacteria culture positive samples [142,143]. Given the difference in functional activity between SP-A1 and SP-A2, as discussed above, it is reasonable to state that the overall functional capacity of SP-A in these patients is significantly altered.

### 4.2. Do SP-A Genetics Play a Role in COVID-19?

Whether the human SP-A1 and SP-A2 variants play a direct or indirect role in COVID-19 remains to be determined. Innate immunity provides the first line of host defense and it is reasonable to think that the level of success or failure of innate immunity determines disease susceptibility and/or severity. The significant body of available data indicates that it is highly likely that the innate immune molecules SP-A1 and SP-A2 play, at the very least, an indirect role in COVID-19 infection and that in this way the SP-A variants may differentially affect disease susceptibility and/or severity or mitigate negative effects due to co-infection of COVID-19 patients with one or more non-SARS-CoV-2 pathogens [144]. It was observed that ~26% of these patients are infected with other pathogens and one of the most common pathogens was respiratory syncytial virus (RSV). SP-A has been shown to enhance RSV clearance [145] and a functional trimeric fragment of SP-A has been shown to be highly efficacious in reducing RSV infection [146]. Moreover, human data have shown an association of SP-A genetic variants with RSV susceptibility [147]. Of direct relevance and interest, is a recent human clinical study where the alveolar macrophage has been implicated in COVID-19 [148]. As has has been extensively investigated in preclinical studies, SP-A variants differentially affect the regulation and function of the macrophage. The authors hypothesized the involvement of innate immunity and that of the macrophage, in particular, in COVID-19 because of the “hyperinflammatory response” many of these patients present with, and because this type of response shares characteristics with the macrophage activation syndrome. A Bruton tyrosine kinase (BTK) inhibitor, that blocks BTK-mediated signaling and activation of the macrophage, was used to treat patients with COVID-19 with promising results as a potential therapeutic strategy. Based on the knowledge from the preclinical studies discussed above, it is likely that the magnitude of the hyperinflammatory response will vary depending on SP-A genotype, which has been shown to differentially affect a number of processes within the alveolus, the alveolar macrophage, and the epithelial cell. Moreover, as already noted, the baseline differences among SP-A variants under normal unprovoked conditions may not have any noticeable negative effect on the overall lung health, but in the face of an insult (i.e., coronavirus or co-infected non-SARS-CoV-2 pathogens) these differences may be critical in determining risk or protection and/or differentially mitigating the downstream dire consequences.

**Figure 6:**
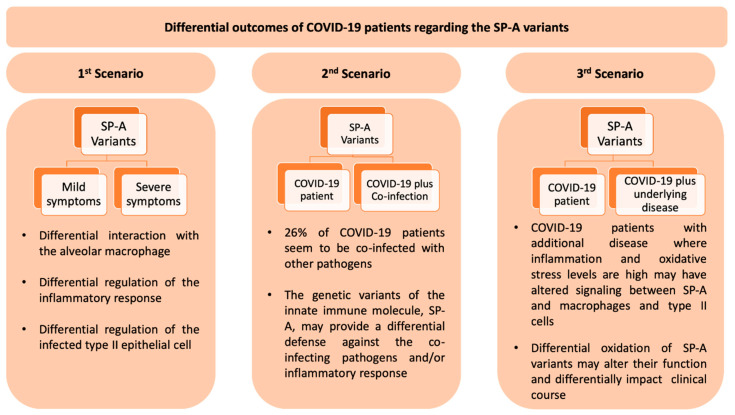
This Figure depicts three scenarios of the potential role of the SP-A innate immune molecules on COVID-19 patients, described in a recent editorial [149] and in part discussed in this review. In the first scenario, individuals with COVID-19 and no co-infection or other underlying disease may experience a differential disease expression from mild to severe based on the SP-A1/SP-A2 genotype. In the second scenario with a co-infecting pathogen, the SP-A genotype may provide a differential host defense against the co-pathogen and an overall differential outcome on COVID-19 patients. In the third scenario patients with underlying disease may experience alterations/dysregulation in inflammatory processes and oxidant levels in the lung. These changes in the lung microenvironment may differentially affect SP-A variant oxidation and thus lung function. In this case the clinical outcome in COVID-19 may vary in ways difficult to predict.

We postulate, based on the available literature, that the SP-A genotype contributes to the relative risk and/or severity of COVID-19 in the presence or absence of co-infection with non-SARS-CoV-2 pathogens and that the macrophage-mediated signaling mechanisms may differentially regulate the “hyperinflammatory response” as a function of the SP-A genotype. In a recent editorial [149] three scenarios were considered as to the potential roles of SP-A variants in Covid-19 and these are depicted in Figure 6. The above postulate provides opportunities for research projects as this is an important and worthwhile research area. Some of these projects may include: (a) preclinical studies using existing SP-A variant-specific animal models to study mechanisms and outcomes in response to coronavirus infection; (b) taking advantage of the current patient availability, human association studies of SP-A genotype and patients infected with coronavirus can be carried out to determine which genotypes are associated with disease susceptibility (risk, protection) and/or disease severity; (c) identification of genetic or epigenetic disease markers to distinguish those with high or low risk; and (d) therapeutic regimens using animal models and information from (a). miRNA therapies may be contemplated based on existing animal data. Understanding the miRNome in health and disease is very important for potential therapies because of its ease of implementation. For example, the use of SP-A variant-regulated miRNAs in therapeutic regimens is far more feasible than using SP-A variants themselves because for the latter it would be much more challenging to produce large amounts of functional SP-A for therapeutic use, as SP-A is highly modified co- and post-translationally [150,151,152,153,154]. Furthermore, the use of trimeric fragments of SP-A instead of the entire molecule could be another therapeutic approach [155].

## 5. Conclusions

The available literature indicates that redox imbalance, lung inflammation, CVDs, aging, and other may contribute to immune dysfunction and the risk of respiratory viral infections, such as SARS-CoV-2. The coordinated managing of a healthy lifestyle status based on pillars such as balanced diet, antioxidant intake, exercise and fasting may be promising ways to attenuate the dire outcomes of this crisis, as these may better position individuals to face ongoing and subsequent waves of COVID-19. Moreover, a healthy lifestyle promoting redox balance may also ensure a better functioning of the innate immune molecule, SP-A, as oxidation of SP-A changes its functional and biochemical properties [111], including its ability to interact with the macrophage [156]. Moreover, O_3_-induced oxidative stress negatively affects macrophage function [157] and regulation [158], either directly or indirectly (via SP-A oxidation) [158]. Because certain plant polyphenols protect SP-A from oxidation [159] and supplementation of antioxidants present in plant foods protect lung function from the ozone-induced negative effects [160,161], it is likely that antioxidant regimens including lifestyle changes, as discussed above, could lead to a well-functioning innate immunity/host defense and consequently better lung health in response to various insults, including COVID-19 infection.

## Abbreviations

WHO	World Health Organization
ROS	Reactive Oxygen Species
O_2_^•−^	superoxide anion
H_2_O_2_	hydrogen peroxide
HO^•^	hydroxyl radical
CAT	catalase
SOD	superoxide dismutase
Trx	thioredoxin
GPx	glutathione peroxidase
GR	glutathione reductase
O_2_	molecular oxygen
ACE2	Angiotensin-converting enzyme 2
AΤ-ΙΙ	Angiotensin 2
AΤ-1,7	Angiotensin 1,7
NADPH oxidase	Nicotinamide adenine dinucleotide phosphate oxidase
SARS-CoV	Severe acute respiratory syndrome coronavirus
SARS-CoV-2	Severe acute respiratory syndrome coronavirus 2
GSH	reduced Glutathione
GSSG	Oxidized Glutathione
H_2_O_2_	hydrogen peroxide
NO	Nitric Oxide
PMNs	Polymorphonuclear cells
IL-6	Interleukin 6
ICU	intensive care unit
COVID-19	Coronavirus disease 2019
COPD	Chronic obstructive pulmonary disease
BMI	Body mass index
CVD	cardiovascular disease
G6PD	Glucose-6-phosphate dehydrogenase deficiency
ECD	endothelial cell dysfunction
NAC	N-acetylcysteine
Nrf2	nuclear factor erythroid 2–related factor 2
KEAP1	Kelch-like ECH-associated protein 1
ARE	Antioxidant response element
ecSOD	Extracellular superoxide dismutase
TBARS	Thiobarbituric acid reactive substances
MDA	Malondialdehyde
mTOR	mammalian target of rapamycin
NF-κB	Nuclear factor-κB
AMPK	AMP-activated protein kinase
SIRT	Sirtuins
FoxO	Forkhead box O
BDNF	Brain-derived neurotrophic factor
TNFα	Tumor necrosis factor alpha
C-RP	c-reactive protein
SP-A	surfactant protein-A
SP-A1	surfactant protein-A1
SP-A2	surfactant protein-A2
OxS	oxidative stress
RSV	respiratory syncytial virus

## References

[1] World Health Organization. www.who.int.

[2] Ray P.D., Huang B.-W., Tsuji Y. (2012). Reactive oxygen species (ROS) homeostasis and redox regulation in cellular signaling. Cell. Signal..

[3] Collin F. (2019). Chemical Basis of Reactive Oxygen Species Reactivity and Involvement in Neurodegenerative Diseases. Int. J. Mol. Sci..

[4] Brieger K., Schiavone S., Miller F.J.J., Krause K.-H. (2012). Reactive oxygen species: From health to disease. Swiss Med. Wkly..

[5] Snezhkina A.V., Kudryavtseva A.V., Kardymon O.L., Savvateeva M.V., Melnikova N.V., Krasnov G.S., Dmitriev A.A. (2019). ROS Generation and Antioxidant Defense Systems in Normal and Malignant Cells. Oxid. Med. Cell. Longev..

[6] Ighodaro O.M., Akinloye O.A. (2018). First line defence antioxidants-superoxide dismutase (SOD), catalase (CAT) and glutathione peroxidase (GPX): Their fundamental role in the entire antioxidant defence grid. Alex. J. Med..

[7] Phelps D.S., Floros J. (1991). Localization of pulmonary surfactant proteins using immunohistochemistry and tissue in situ hybridization. Exp. Lung Res..

[8] Wang G., Umstead T.M., Phelps D.S., Al-Mondhiry H., Floros J. (2002). The effect of ozone exposure on the ability of human surfactant protein a variants to stimulate cytokine production. Environ. Health Perspect..

[9] Mikerov A.N., Wang G., Umstead T.M., Zacharatos M., Thomas N.J., Phelps D.S., Floros J. (2007). Surfactant protein A2 (SP-A2) variants expressed in CHO cells stimulate phagocytosis of Pseudomonas aeruginosa more than do SP-A1 variants. Infect. Immun..

[10] Mikerov A.N., Umstead T.M., Gan X., Huang W., Guo X., Wang G., Phelps D.S., Floros J. (2008). Impact of ozone exposure on the phagocytic activity of human surfactant protein A (SP-A) and SP-A variants. Am. J. Physiol. Lung Cell. Mol. Physiol..

[11] Thorenoor N., Umstead T.M., Zhang X., Phelps D.S., Floros J. (2018). Survival of Surfactant Protein-A1 and SP-A2 Transgenic Mice After Klebsiella pneumoniae Infection, Exhibits Sex-, Gene-, and Variant Specific Differences; Treatment With Surfactant Protein Improves Survival. Front. Immunol..

[12] Thorenoor N., Zhang X., Umstead T.M., Scott Halstead E., Phelps D.S., Floros J. (2018). Differential effects of innate immune variants of surfactant protein-A1 (SFTPA1) and SP-A2 (SFTPA2) in airway function after Klebsiella pneumoniae infection and sex differences. Respir. Res..

[13] Wan Y., Shang J., Graham R., Baric R.S., Li F. (2020). Receptor Recognition by the Novel Coronavirus from Wuhan: An Analysis Based on Decade-Long Structural Studies of SARS Coronavirus. J. Virol..

[14] Andersen K.G., Rambaut A., Lipkin W.I., Holmes E.C., Garry R.F. (2020). The proximal origin of SARS-CoV-2. Nat. Med..

[15] Wrapp D., Wang N., Corbett K.S., Goldsmith J.A., Hsieh C.-L., Abiona O., Graham B.S., McLellan J.S. (2020). Cryo-EM structure of the 2019-nCoV spike in the prefusion conformation. Science.

[16] Letko M., Marzi A., Munster V. (2020). Functional assessment of cell entry and receptor usage for SARS-CoV-2 and other lineage B betacoronaviruses. Nat. Microbiol..

[17] Keidar S., Kaplan M., Gamliel-Lazarovich A. (2007). ACE2 of the heart: From angiotensin I to angiotensin (1-7). Cardiovasc. Res..

[18] Wang W., McKinnie S.M.K., Farhan M., Paul M., McDonald T., McLean B., Llorens-Cortes C., Hazra S., Murray A.G., Vederas J.C. (2016). Angiotensin-Converting Enzyme 2 Metabolizes and Partially Inactivates Pyr-Apelin-13 and Apelin-17: Physiological Effects in the Cardiovascular System. Hypertension.

[19] Donoghue M., Hsieh F., Baronas E., Godbout K., Gosselin M., Stagliano N., Donovan M., Woolf B., Robison K., Jeyaseelan R. (2000). A novel angiotensin-converting enzyme-related carboxypeptidase (ACE2) converts angiotensin I to angiotensin 1-9. Circ. Res..

[20] Polizio A.H., Gironacci M.M., Tomaro M.L., Peña C. (2007). Angiotensin-(1-7) blocks the angiotensin II-stimulated superoxide production. Pharmacol. Res..

[21] Lovren F., Pan Y., Quan A., Teoh H., Wang G., Shukla P.C., Levitt K.S., Oudit G.Y., Al-Omran M., Stewart D.J. (2008). Angiotensin converting enzyme-2 confers endothelial protection and attenuates atherosclerosis. Am. J. Physiol. Heart Circ. Physiol..

[22] Zhang Y.-H., Zhang Y., Dong X.-F., Hao Q.-Q., Zhou X.-M., Yu Q.-T., Li S.-Y., Chen X., Tengbeh A.F., Dong B. (2015). ACE2 and Ang-(1-7) protect endothelial cell function and prevent early atherosclerosis by inhibiting inflammatory response. Inflamm. Res..

[23] Stanhewicz A.E., Alexander L.M. (2020). Local angiotensin-(1-7) administration improves microvascular endothelial function in women who have had preeclampsia. Am. J. Physiol. Regul. Integr. Comp. Physiol..

[24] Liu J., Liu Y., Xiang P., Pu L., Xiong H., Li C., Zhang M., Tan J., Xu Y., Song R. (2020). Neutrophil-to-lymphocyte ratio predicts critical illness patients with 2019 coronavirus disease in the early stage. J. Transl. Med..

[25] Meitzler J.L., Antony S., Wu Y., Juhasz A., Liu H., Jiang G., Lu J., Roy K., Doroshow J.H. (2014). NADPH oxidases: A perspective on reactive oxygen species production in tumor biology. Antioxid. Redox Signal..

[26] Madamanchi N.R., Hakim Z.S., Runge M.S. (2005). Oxidative stress in atherogenesis and arterial thrombosis: The disconnect between cellular studies and clinical outcomes. J. Thromb. Haemost..

[27] Wen H., Gwathmey J.K., Xie L.-H. (2012). Oxidative stress-mediated effects of angiotensin II in the cardiovascular system. World J. Hypertens..

[28] Prompetchara E., Ketloy C., Palaga T. (2020). Immune responses in COVID-19 and potential vaccines: Lessons learned from SARS and MERS epidemic. Asian Pac. J. Allergy Immunol..

[29] Nguyen G.T., Green E.R., Mecsas J. (2017). Neutrophils to the ROScue: Mechanisms of NADPH Oxidase Activation and Bacterial Resistance. Front. Cell. Infect. Microbiol..

[30] Richardson S., Hirsch J.S., Narasimhan M., Crawford J.M., McGinn T., Davidson K.W., Barnaby D.P., Becker L.B., Chelico J.D., Cohen S.L. (2020). Presenting Characteristics, Comorbidities, and Outcomes Among 5700 Patients Hospitalized With COVID-19 in the New York City Area. JAMA.

[31] Tan B.L., Norhaizan M.E., Liew W.-P.-P. (2018). Nutrients and Oxidative Stress: Friend or Foe?. Oxid. Med. Cell. Longev..

[32] Hadi H.A.R., Carr C.S., Al Suwaidi J. (2005). Endothelial dysfunction: Cardiovascular risk factors, therapy, and outcome. Vasc. Health Risk Manag..

[33] Bayraktutan U. (2002). Free radicals, diabetes and endothelial dysfunction. Diabetes Obes. Metab..

[34] SHI Y., Vanhoutte P.M. (2009). Reactive oxygen-derived free radicals are key to the endothelial dysfunction of diabetes. J. Diabetes.

[35] Ma X.L., Gao F., Nelson A.H., Lopez B.L., Christopher T.A., Yue T.L., Barone F.C. (2001). Oxidative inactivation of nitric oxide and endothelial dysfunction in stroke-prone spontaneous hypertensive rats. J. Pharmacol. Exp. Ther..

[36] Loscalzo J. (2002). Oxidative stress in endothelial cell dysfunction and thrombosis. Pathophysiol. Haemost. Thromb..

[37] Mangge H., Becker K., Fuchs D., Gostner J.M. (2014). Antioxidants, inflammation and cardiovascular disease. World J. Cardiol..

[38] Nasi A., McArdle S., Gaudernack G., Westman G., Melief C., Rockberg J., Arens R., Kouretas D., Sjölin J., Mangsbo S. (2020). Reactive oxygen species as an initiator of toxic innate immune responses in retort to SARS-CoV-2 in an ageing population, consider N-acetylcysteine as early therapeutic intervention. Toxicol. Rep..

[39] Veskoukis A.S., Preedy V.R.B.T.-P. (2020). Chapter 8—Redox Signaling and Antioxidant Defense in Pathogenic Microorganisms: A Link to Disease and Putative Therapy.

[40] Halasi M., Wang M., Chavan T.S., Gaponenko V., Hay N., Gartel A.L. (2013). ROS inhibitor N-acetyl-L-cysteine antagonizes the activity of proteasome inhibitors. Biochem. J..

[41] Smithers G.W. (2008). Whey and whey proteins—From ‘gutter-to-gold’. Int. Dairy J..

[42] Veskoukis A.S., Tsatsakis A.M., Kouretas D. (2012). Dietary oxidative stress and antioxidant defense with an emphasis on plant extract administration. Cell Stress Chaperones.

[43] Marshall K. (2004). Therapeutic applications of whey protein. Altern. Med. Rev..

[44] Veskoukis A., Kerasioti E., Priftis A., Kouka P., Spanidis Y., Makri S., Kouretas D. (2019). A battery of translational biomarkers for the assessment of the in vitro and in vivo antioxidant action of plant polyphenolic compounds: The biomarker issue. Curr. Opin. Toxicol..

[45] Kerasioti E., Stagos D., Tzimi A., Kouretas D. (2016). Increase in antioxidant activity by sheep/goat whey protein through nuclear factor-like 2 (Nrf2) is cell type dependent. Food Chem. Toxicol..

[46] Kerasioti E., Stagos D., Georgatzi V., Bregou E., Priftis A., Kafantaris I., Kouretas D. (2016). Antioxidant Effects of Sheep Whey Protein on Endothelial Cells. Oxid. Med. Cell. Longev..

[47] Kerasioti E., Stagos D., Priftis A., Aivazidis S., Tsatsakis A.M., Hayes A.W., Kouretas D. (2014). Antioxidant effects of whey protein on muscle C2C12 cells. Food Chem..

[48] Kerasioti E., Stagos D., Jamurtas A., Kiskini A., Koutedakis Y., Goutzourelas N., Pournaras S., Tsatsakis A.M., Kouretas D. (2013). Anti-inflammatory effects of a special carbohydrate-whey protein cake after exhaustive cycling in humans. Food Chem. Toxicol..

[49] Kerasioti E., Stagos D., Tsatsakis A.M., Spandidos D.A., Taitzoglou I., Kouretas D. (2018). Effects of sheep/goat whey protein dietary supplementation on the redox status of rats. Mol. Med. Rep..

[50] Kerasioti E., Veskoukis A., Virgiliou C., Theodoridis G., Taitzoglou I., Kouretas D. (2019). The Strong Antioxidant Sheep/Goat Whey Protein Protects Against mTOR Overactivation in Rats: A Mode of Action Mimicking Fasting. Antioxidants.

[51] Draganidis D., Karagounis L.G., Athanailidis I., Chatzinikolaou A., Jamurtas A.Z., Fatouros I.G. (2016). Inflammaging and Skeletal Muscle: Can Protein Intake Make a Difference?. J. Nutr..

[52] Goutzourelas N., Stagos D., Spanidis Y., Liosi M., Apostolou A., Priftis A., Haroutounian S., Spandidos D.A., Tsatsakis A.M., Kouretas D. (2015). Polyphenolic composition of grape stem extracts affects antioxidant activity in endothelial and muscle cells. Mol. Med. Rep..

[53] Goutzourelas N., Stagos D., Demertzis N., Mavridou P., Karterolioti H., Georgadakis S., Kerasioti E., Aligiannis N., Skaltsounis L., Statiri A. (2014). Effects of polyphenolic grape extract on the oxidative status of muscle and endothelial cells. Hum. Exp. Toxicol..

[54] Kouka P., Priftis A., Stagos D., Angelis A., Stathopoulos P., Xinos N., Skaltsounis A.-L., Mamoulakis C., Tsatsakis A.M., Spandidos D.A. (2017). Assessment of the antioxidant activity of an olive oil total polyphenolic fraction and hydroxytyrosol from a Greek Olea europea variety in endothelial cells and myoblasts. Int. J. Mol. Med..

[55] Makri S., Kafantaris I., Savva S., Ntanou P., Stagos D., Argyroulis I., Kotsampasi B., Christodoulou V., Gerasopoulos K., Petrotos K. (2018). Novel Feed Including Olive Oil Mill Wastewater Bioactive Compounds Enhanced the Redox Status of Lambs. In Vivo.

[56] Kafantaris I., Kotsampasi B., Christodoulou V., Kokka E., Kouka P., Terzopoulou Z., Gerasopoulos K., Stagos D., Mitsagga C., Giavasis I. (2017). Grape pomace improves antioxidant capacity and faecal microflora of lambs. J. Anim. Physiol. Anim. Nutr. Berl..

[57] Makri S., Kafantaris I., Stagos D., Chamokeridou T., Petrotos K., Gerasopoulos K., Mpesios A., Goutzourelas N., Kokkas S., Goulas P. (2017). Novel feed including bioactive compounds from winery wastes improved broilers’ redox status in blood and tissues of vital organs. Food Chem. Toxicol..

[58] Kafantaris I., Stagos D., Kotsampasi B., Hatzis A., Kypriotakis A., Gerasopoulos K., Makri S., Goutzourelas N., Mitsagga C., Giavasis I. (2018). Grape pomace improves performance, antioxidant status, fecal microbiota and meat quality of piglets. Animal.

[59] Gerasopoulos K., Stagos D., Kokkas S., Petrotos K., Kantas D., Goulas P., Kouretas D. (2015). Feed supplemented with byproducts from olive oil mill wastewater processing increases antioxidant capacity in broiler chickens. Food Chem. Toxicol..

[60] Matthaiou C.M., Goutzourelas N., Stagos D., Sarafoglou E., Jamurtas A., Koulocheri S.D., Haroutounian S.A., Tsatsakis A.M., Kouretas D. (2014). Pomegranate juice consumption increases GSH levels and reduces lipid and protein oxidation in human blood. Food Chem. Toxicol..

[61] Ma Q. (2013). Role of nrf2 in oxidative stress and toxicity. Annu. Rev. Pharmacol. Toxicol..

[62] Zhang H., Davies K.J.A., Forman H.J. (2015). Oxidative stress response and Nrf2 signaling in aging. Free Radic. Biol. Med..

[63] Zhou Y., Jiang Z., Lu H., Xu Z., Tong R., Shi J., Jia G. (2019). Recent Advances of Natural Polyphenols Activators for Keap1-Nrf2 Signaling Pathway. Chem. Biodivers..

[64] Raghunath A., Sundarraj K., Nagarajan R., Arfuso F., Bian J., Kumar A.P., Sethi G., Perumal E. (2018). Antioxidant response elements: Discovery, classes, regulation and potential applications. Redox Biol..

[65] Fan E., Brodie D., Slutsky A.S. (2018). Acute Respiratory Distress Syndrome: Advances in Diagnosis and Treatment. JAMA.

[66] Gordon L.A., Morrison E.Y., McGrowder D.A., Young R., Fraser Y.T.P., Zamora E.M., Alexander-Lindo R.L., Irving R.R. (2008). Effect of exercise therapy on lipid profile and oxidative stress indicators in patients with type 2 diabetes. BMC Complement. Altern. Med..

[67] de Oliveira V.N., Bessa A., Jorge M.L.M.P., Oliveira R.J.D.S., de Mello M.T., De Agostini G.G., Jorge P.T., Espindola F.S. (2012). The effect of different training programs on antioxidant status, oxidative stress, and metabolic control in type 2 diabetes. Appl. Physiol. Nutr. Metab. Physiol. Appl. Nutr. Metab..

[68] Azizbeigi K., Azarbayjani M.A., Peeri M., Agha-alinejad H., Stannard S. (2013). The effect of progressive resistance training on oxidative stress and antioxidant enzyme activity in erythrocytes in untrained men. Int. J. Sport Nutr. Exerc. Metab..

[69] Karabulut A.B., Kafkas M.E., Kafkas A.S., Onal Y., Kiran T.R. (2013). The effect of regular exercise and massage on oxidant and antioxidant parameters. Indian J. Physiol. Pharmacol..

[70] Johnson M.L., Irving B.A., Lanza I.R., Vendelbo M.H., Konopka A.R., Robinson M.M., Henderson G.C., Klaus K.A., Morse D.M., Heppelmann C. (2015). Differential Effect of Endurance Training on Mitochondrial Protein Damage, Degradation, and Acetylation in the Context of Aging. J. Gerontol. A Biol. Sci. Med. Sci..

[71] Mitranun W., Deerochanawong C., Tanaka H., Suksom D. (2014). Continuous vs interval training on glycemic control and macro- and microvascular reactivity in type 2 diabetic patients. Scand. J. Med. Sci. Sports.

[72] Linke A., Adams V., Schulze P.C., Erbs S., Gielen S., Fiehn E., Möbius-Winkler S., Schubert A., Schuler G., Hambrecht R. (2005). Antioxidative effects of exercise training in patients with chronic heart failure: Increase in radical scavenger enzyme activity in skeletal muscle. Circulation.

[73] Onur E., Kabaroğlu C., Günay O., Var A., Yilmaz O., Dündar P., Tikiz C., Güvenç Y., Yüksel H. (2011). The beneficial effects of physical exercise on antioxidant status in asthmatic children. Allergol. Immunopathol. Madr..

[74] Beck D.T., Martin J.S., Casey D.P., Braith R.W. (2014). Exercise training improves endothelial function in resistance arteries of young prehypertensives. J. Hum. Hypertens..

[75] Soares J.P., Silva A.M., Oliveira M.M., Peixoto F., Gaivão I., Mota M.P. (2015). Effects of combined physical exercise training on DNA damage and repair capacity: Role of oxidative stress changes. Age Dord..

[76] Di Francesco A., Di Germanio C., Bernier M., de Cabo R. (2018). A time to fast. Science.

[77] Prentice A.M., Whitehead R.G., Roberts S.B., Paul A.A. (1981). Long-term energy balance in child-bearing Gambian women. Am. J. Clin. Nutr..

[78] Venegas-Borsellino C., Martindale R.G. (2018). From Religion to Secularism: The Benefits of Fasting. Curr. Nutr. Rep..

[79] de Toledo F.W., Grundler F., Bergouignan A., Drinda S., Michalsen A. (2019). Safety, health improvement and well-being during a 4 to 21-day fasting period in an observational study including 1422 subjects. PLoS ONE.

[80] de Toledo F.W., Buchinger A., Burggrabe H., Hölz G., Kuhn C., Lischka E., Lischka N., Lützner H., May W., Ritzmann-Widderich M. (2013). Fasting therapy—An expert panel update of the 2002 consensus guidelines. Complement. Med. Res..

[81] Mesnage R., Grundler F., Schwiertz A., Le Maho Y., de Toledo F.W. (2019). Changes in human gut microbiota composition are linked to the energy metabolic switch during 10 d of Buchinger fasting. J. Nutr. Sci..

[82] Cherif A., Roelands B., Meeusen R., Chamari K. (2016). Effects of Intermittent Fasting, Caloric Restriction, and Ramadan Intermittent Fasting on Cognitive Performance at Rest and During Exercise in Adults. Sports Med..

[83] Mattson M.P., Longo V.D., Harvie M. (2017). Impact of intermittent fasting on health and disease processes. Ageing Res. Rev..

[84] Patterson R.E., Sears D.D. (2017). Metabolic Effects of Intermittent Fasting. Annu. Rev. Nutr..

[85] Tinsley G.M., La Bounty P.M. (2015). Effects of intermittent fasting on body composition and clinical health markers in humans. Nutr. Rev..

[86] Antoni R., Johnston K.L., Collins A.L., Robertson M.D. (2017). Effects of intermittent fasting on glucose and lipid metabolism. Proc. Nutr. Soc..

[87] Vázquez-Medina J.P., Popovich I., Thorwald M.A., Viscarra J.A., Rodriguez R., Sonanez-Organis J.G., Lam L., Peti-Peterdi J., Nakano D., Nishiyama A. (2013). Angiotensin receptor-mediated oxidative stress is associated with impaired cardiac redox signaling and mitochondrial function in insulin-resistant rats. Am. J. Physiol. Circ. Physiol..

[88] Vázquez-Medina J.P., Soñanez-Organis J.G., Rodriguez R., Viscarra J.A., Nishiyama A., Crocker D.E., Ortiz R.M. (2013). Prolonged fasting activates Nrf2 in post-weaned elephant seals. J. Exp. Biol..

[89] de Toledo F.W., Grundler F., Goutzourelas N., Tekos F., Vassi E., Mesnage R., Kouretas D. (2020). Influence of Long-Term Fasting on Blood Redox Status in Humans. Antioxidants.

[90] Anton S.D., Moehl K., Donahoo W.T., Marosi K., Lee S.A., Mainous A.G., Leeuwenburgh C., Mattson M.P. (2018). Flipping the Metabolic Switch: Understanding and Applying the Health Benefits of Fasting. Obes. Silver Spring.

[91] Mattson M.P., Moehl K., Ghena N., Schmaedick M., Cheng A. (2018). Intermittent metabolic switching, neuroplasticity and brain health. Nat. Rev. Neurosci..

[92] Fontana L., Partridge L., Longo V.D. (2010). Extending healthy life span—From yeast to humans. Science.

[93] López-Otín C., Blasco M.A., Partridge L., Serrano M., Kroemer G. (2013). The hallmarks of aging. Cell.

[94] Madkour M.I., El-Serafi A.T., Jahrami H.A., Sherif N.M., Hassan R.E., Awadallah S., Faris M.A.-I.E. (2019). Ramadan diurnal intermittent fasting modulates SOD2, TFAM, Nrf2, and sirtuins (SIRT1, SIRT3) gene expressions in subjects with overweight and obesity. Diabetes Res. Clin. Pract..

[95] Halberg N., Henriksen M., Söderhamn N., Stallknecht B., Ploug T., Schjerling P., Dela F. (2005). Effect of intermittent fasting and refeeding on insulin action in healthy men. J. Appl. Physiol..

[96] Johnson J.B., Summer W., Cutler R.G., Martin B., Hyun D.-H., Dixit V.D., Pearson M., Nassar M., Telljohann R., Maudsley S. (2007). Alternate day calorie restriction improves clinical findings and reduces markers of oxidative stress and inflammation in overweight adults with moderate asthma. Free Radic. Biol. Med..

[97] Varady K.A., Bhutani S., Klempel M.C., Kroeger C.M., Trepanowski J.F., Haus J.M., Hoddy K.K., Calvo Y. (2013). Alternate day fasting for weight loss in normal weight and overweight subjects: A randomized controlled trial. Nutr. J..

[98] Asemi Z., Samimi M., Taghizadeh M., Esmaillzadeh A. (2015). Effects of Ramadan Fasting on Glucose Homeostasis, Lipid Profiles, Inflammation and Oxidative Stress in Women with Polycystic Ovary Syndrome in Kashan, Iran. Arch. Iran. Med..

[99] Moro T., Tinsley G., Bianco A., Marcolin G., Pacelli Q.F., Battaglia G., Palma A., Gentil P., Neri M., Paoli A. (2016). Effects of eight weeks of time-restricted feeding (16/8) on basal metabolism, maximal strength, body composition, inflammation, and cardiovascular risk factors in resistance-trained males. J. Transl. Med..

[100] Faris M.A.-I.E., Kacimi S., Al-Kurd R.A., Fararjeh M.A., Bustanji Y.K., Mohammad M.K., Salem M.L. (2012). Intermittent fasting during Ramadan attenuates proinflammatory cytokines and immune cells in healthy subjects. Nutr. Res..

[101] Aliasghari F., Izadi A., Gargari B.P., Ebrahimi S. (2017). The Effects of Ramadan Fasting on Body Composition, Blood Pressure, Glucose Metabolism, and Markers of Inflammation in NAFLD Patients: An Observational Trial. J. Am. Coll. Nutr..

[102] Kroeger C.M., Klempel M.C., Bhutani S., Trepanowski J.F., Tangney C.C., Varady K.A. (2012). Improvement in coronary heart disease risk factors during an intermittent fasting/calorie restriction regimen: Relationship to adipokine modulations. Nutr. Metab. Lond..

[103] Karinch A.M., Floros J. (1995). 5’ splicing and allelic variants of the human pulmonary surfactant protein A genes. Am. J. Respir. Cell Mol. Biol..

[104] Hoover R.R., Floros J. (1998). Organization of the human SP-A and SP-D loci at 10q22-q23. Physical and radiation hybrid mapping reveal gene order and orientation. Am. J. Respir. Cell Mol. Biol..

[105] Bruns G., Stroh H., Veldman G.M., Latt S.A., Floros J. (1987). The 35 kd pulmonary surfactant-associated protein is encoded on chromosome 10. Hum. Genet..

[106] Floros J., Hoover R.R. (1998). Genetics of the hydrophilic surfactant proteins A and D. Biochim. Biophys. Acta.

[107] Floros J., Wang G., Lin Z. (2005). Genetic diversity of human SP-A, a molecule with innate host defense and surfactant-related functions; characteristics, primary function, and significance. Curr. Pharm..

[108] Floros J., Wang G., Mikerov A.N. (2009). Genetic complexity of the human innate host defense molecules, surfactant protein A1 (SP-A1) and SP-A2--impact on function. Crit. Rev. Eukaryot Gene Expr..

[109] DiAngelo S., Lin Z., Wang G., Phillips S., Ramet M., Luo J., Floros J. (1999). Novel, non-radioactive, simple and multiplex PCR-cRFLP methods for genotyping human SP-A and SP-D marker alleles. Dis. Markers.

[110] Voss T., Melchers K., Scheirle G., Schäfer K.P. (1991). Structural comparison of recombinant pulmonary surfactant protein SP-A derived from two human coding sequences: Implications for the chain composition of natural human SP-A. Am. J. Respir. Cell Mol. Biol..

[111] Wang G., Bates-Kenney S.R., Tao J.-Q., Phelps D.S., Floros J. (2004). Differences in biochemical properties and in biological function between human SP-A1 and SP-A2 variants, and the impact of ozone-induced oxidation. Biochemistry.

[112] Floros J., Wang G. (2001). A point of view: Quantitative and qualitative imbalance in disease pathogenesis; pulmonary surfactant protein A genetic variants as a model. Comp. Biochem. Physiol. A Mol. Integr. Physiol..

[113] Wang G., Myers C., Mikerov A., Floros J. (2007). Effect of cysteine 85 on biochemical properties and biological function of human surfactant protein A variants. Biochemistry.

[114] Karinch A.M., Floros J. (1995). Translation in vivo of 5’ untranslated-region splice variants of human surfactant protein-A. Biochem. J..

[115] Silveyra P., Wang G., Floros J. (2010). Human SP-A1 (SFTPA1) variant-specific 3’ UTRs and poly(A) tail differentially affect the in vitro translation of a reporter gene. Am. J. Physiol. Lung Cell. Mol. Physiol..

[116] Silveyra P., DiAngelo S.L., Floros J. (2014). An 11-nt sequence polymorphism at the 3’UTR of human SFTPA1 and SFTPA2 gene variants differentially affect gene expression levels and miRNA regulation in cell culture. Am. J. Physiol. Lung Cell. Mol. Physiol..

[117] García-Verdugo I., Wang G., Floros J., Casals C. (2002). Structural analysis and lipid-binding properties of recombinant human surfactant protein a derived from one or both genes. Biochemistry.

[118] Wang G., Taneva S., Keough K.M.W., Floros J. (2007). Differential effects of human SP-A1 and SP-A2 variants on phospholipid monolayers containing surfactant protein B. Biochim. Biophys. Acta.

[119] Phelps D.S., Umstead T.M., Silveyra P., Hu S., Wang G., Floros J. (2013). Differences in the alveolar macrophage proteome in transgenic mice expressing human SP-A1 and SP-A2. J. Proteom. Genom. Res..

[120] Lopez-Rodriguez E., Pascual A., Arroyo R., Floros J., Perez-Gil J. (2016). Human Pulmonary Surfactant Protein SP-A1 Provides Maximal Efficiency of Lung Interfacial Films. Biophys. J..

[121] Tsotakos N., Phelps D.S., Yengo C.M., Chinchilli V.M., Floros J. (2016). Single-cell analysis reveals differential regulation of the alveolar macrophage actin cytoskeleton by surfactant proteins A1 and A2: Implications of sex and aging. Biol. Sex Differ..

[122] Noutsios G.T., Thorenoor N., Zhang X., Phelps D.S., Umstead T.M., Durrani F., Floros J. (2017). SP-A2 contributes to miRNA-mediated sex differences in response to oxidative stress: Pro-inflammatory, anti-apoptotic, and anti-oxidant pathways are involved. Biol. Sex Differ..

[123] Wang G., Umstead T.M., Hu S., Mikerov A.N., Phelps D.S., Floros J. (2019). Differential Effects of Human SP-A1 and SP-A2 on the BAL Proteome and Signaling Pathways in Response to Klebsiella pneumoniae and Ozone Exposure. Front. Immunol..

[124] Thorenoor N., Kawasawa Y.I., Gandhi C.K., Zhang X., Floros J. (2019). Differential Impact of Co-expressed SP-A1/SP-A2 Protein on AM miRNome; Sex Differences. Front. Immunol..

[125] Noutsios G.T., Thorenoor N., Zhang X., Phelps D.S., Umstead T.M., Durrani F., Floros J. (2019). Major Effect of Oxidative Stress on the Male, but Not Female, SP-A1 Type II Cell miRNome. Front. Immunol..

[126] Phelps D.S., Umstead T.M., Floros J. (2014). Sex differences in the acute in vivo effects of different human SP-A variants on the mouse alveolar macrophage proteome. J. Proteom..

[127] Nalian A., Umstead T.M., Yang C.-H., Silveyra P., Thomas N.J., Floros J., McCormack F.X., Chroneos Z.C. (2019). Structural and Functional Determinants of Rodent and Human Surfactant Protein A: A Synthesis of Binding and Computational Data. Front. Immunol..

[128] Floros J., Thomas N., Nakos G., Papathanasiou A. (2009). Genetic variations of surfactant proteins and lung injury. Surfactant Pathogenesis and Treatment of Lung Disease.

[129] Silveyra P., Floros J. (2012). Genetic variant associations of human SP-A and SP-D with acute and chronic lung injury. Front. Biosci..

[130] Wang G., Phelps D.S., Umstead T.M., Floros J. (2000). Human SP-A protein variants derived from one or both genes stimulate TNF-alpha production in the THP-1 cell line. Am. J. Physiol. Lung Cell. Mol. Physiol..

[131] Mikerov A.N., Umstead T.M., Huang W., Liu W., Phelps D.S., Floros J. (2005). SP-A1 and SP-A2 variants differentially enhance association of Pseudomonas aeruginosa with rat alveolar macrophages. Am. J. Physiol. Lung Cell. Mol. Physiol..

[132] Thorenoor N., Kawasawa Y.I., Ghandi C.K., Floros J. (2020). Sex-specific regulation of gene expression networks by surfactant protein A (SP-A) variants in alveolar macrophages in response to Klebsiella pneumoniae. Front. Immunol..

[133] D’Ovidio F., Floros J., Aramini B., Lederer D., DiAngelo S.L., Arcasoy S., Sonett J.R., Robbins H., Shah L., Costa J. (2020). Donor surfactant protein A2 polymorphism and lung transplant survival. Eur. Respir. J..

[134] D’Ovidio F., Kaneda H., Chaparro C., Mura M., Lederer D., Di Angelo S., Takahashi H., Gutierrez C., Hutcheon M., Singer L.G. (2013). Pilot study exploring lung allograft surfactant protein A (SP-A) expression in association with lung transplant outcome. Am. J. Transpl..

[135] Karinch A.M., Deiter G., Ballard P.L., Floros J. (1998). Regulation of expression of human SP-A1 and SP-A2 genes in fetal lung explant culture. Biochim. Biophys. Acta.

[136] Wang G., Guo X., Floros J. (2003). Human SP-A 3’-UTR variants mediate differential gene expression in basal levels and in response to dexamethasone. Am. J. Physiol. Lung Cell. Mol. Physiol..

[137] Wang G., Guo X., Floros J. (2005). Differences in the translation efficiency and mRNA stability mediated by 5’-UTR splice variants of human SP-A1 and SP-A2 genes. Am. J. Physiol. Lung Cell. Mol. Physiol..

[138] Wang G., Guo X., Silveyra P., Kimball S.R., Floros J. (2009). Cap-independent translation of human SP-A 5’-UTR variants: A double-loop structure and cis-element contribution. Am. J. Physiol. Lung Cell. Mol. Physiol..

[139] Noutsios G.T., Silveyra P., Bhatti F., Floros J. (2013). Exon B of human surfactant protein A2 mRNA, alone or within its surrounding sequences, interacts with 14-3-3; role of cis-elements and secondary structure. Am. J. Physiol. Lung Cell. Mol. Physiol..

[140] Noutsios G.T., Ghattas P., Bennett S., Floros J. (2015). 14-3-3 isoforms bind directly exon B of the 5’-UTR of human surfactant protein A2 mRNA. Am. J. Physiol. Lung Cell. Mol. Physiol..

[141] Aramini B., Geraghty P., Lederer D.J., Costa J., DiAngelo S.L., Floros J., D’Ovidio F. (2019). Surfactant protein A and D polymorphisms and methylprednisolone pharmacogenetics in donor lungs. J. Thorac. Cardiovasc. Surg..

[142] Tagaram H.R.S., Wang G., Umstead T.M., Mikerov A.N., Thomas N.J., Graff G.R., Hess J.C., Thomassen M.J., Kavuru M.S., Phelps D.S. (2007). Characterization of a human surfactant protein A1 (SP-A1) gene-specific antibody; SP-A1 content variation among individuals of varying age and pulmonary health. Am. J. Physiol. Lung Cell. Mol. Physiol..

[143] Wang Y., Voelker D.R., Lugogo N.L., Wang G., Floros J., Ingram J.L., Chu H.W., Church T.D., Kandasamy P., Fertel D. (2011). Surfactant protein A is defective in abrogating inflammation in asthma. Am. J. Physiol. Lung Cell. Mol. Physiol..

[144] Kim D., Quinn J., Pinsky B., Shah N.H., Brown I. (2020). Rates of Co-infection Between SARS-CoV-2 and Other Respiratory Pathogens. JAMA.

[145] LeVine A.M., Gwozdz J., Stark J., Bruno M., Whitsett J., Korfhagen T. (1999). Surfactant protein-A enhances respiratory syncytial virus clearance in vivo. J. Clin. Investig..

[146] Watson A., Kronqvist N., Spalluto C.M., Griffiths M., Staples K.J., Wilkinson T., Holmskov U., Sorensen G.L., Rising A., Johansson J. (2017). Novel expression of a functional trimeric fragment of human SP-A with efficacy in neutralisation of RSV. Immunobiology.

[147] Thomas N.J., Fan R., Diangelo S., Hess J.C., Floros J. (2007). Haplotypes of the surfactant protein genes A and D as susceptibility factors for the development of respiratory distress syndrome. Acta Paediatr..

[148] Roschewski M., Lionakis M.S., Sharman J.P., Roswarski J., Goy A., Monticelli M.A., Roshon M., Wrzesinski S.H., Desai J.V., Zarakas M.A. (2020). Inhibition of Bruton tyrosine kinase in patients with severe COVID-19. Sci. Immunol..

[149] Floros J., Phelps D.S. (2020). Is the role of lung innate immune molecules, SP-A1 and SP-A2, and of the alveolar macrophage being overlooked in COVID-19 diverse outcomes?. Pneumon.

[150] Phelps D.S., Floros J., Taeusch H.W.J. (1986). Post-translational modification of the major human surfactant-associated proteins. Biochem. J..

[151] Floros J., Steinbrink R., Jacobs K., Phelps D., Kriz R., Recny M., Sultzman L., Jones S., Taeusch H.W., Frank H.A. (1986). Isolation and characterization of cDNA clones for the 35-kDa pulmonary surfactant-associated protein. J. Biol. Chem..

[152] Phelps D.S., Floros J. (1988). Proline hydroxylation alters the electrophoretic mobility of pulmonary surfactant-associated protein A. Electrophoresis.

[153] Floros J., Phelps D.S., Taeusch H.W. (1985). Biosynthesis and in vitro translation of the major surfactant-associated protein from human lung. J. Biol. Chem..

[154] Floros J., Phelps D.S., Kourembanas S., Taeusch H.W. (1986). Primary translation products, biosynthesis, and tissue specificity of the major surfactant protein in rat. J. Biol. Chem..

[155] Watson A., Sørensen G.L., Holmskov U., Whitwell H.J., Madsen J., Clark H. (2020). Generation of novel trimeric fragments of human SP-A and SP-D after recombinant soluble expression in *E. coli*. Immunobiology.

[156] Oosting R.S., Van Iwaarden J.F., Van Bree L., Verhoef J., Van Golde L.M., Haagsman H.P. (1992). Exposure of surfactant protein A to ozone in vitro and in vivo impairs its interactions with alveolar cells. Am. J. Physiol..

[157] Mikerov A.N., Haque R., Gan X., Guo X., Phelps D.S., Floros J. (2008). Ablation of SP-A has a negative impact on the susceptibility of mice to Klebsiella pneumoniae infection after ozone exposure: Sex differences. Respir. Res..

[158] Janic B., Umstead T.M., Phelps D.S., Floros J. (2005). Modulatory effects of ozone on THP-1 cells in response to SP-A stimulation. Am. J. Physiol. Lung Cell. Mol. Physiol..

[159] Stagos D., Umstead T.M., Phelps D.S., Skaltsounis L., Haroutounian S., Floros J., Kouretas D. (2007). Inhibition of ozone-induced SP-A oxidation by plant polyphenols. Free Radic. Res..

[160] Samet J.M., Hatch G.E., Horstman D., Steck-Scott S., Arab L., Bromberg P.A., Levine M., McDonnell W.F., Devlin R.B. (2001). Effect of antioxidant supplementation on ozone-induced lung injury in human subjects. Am. J. Respir. Crit. Care Med..

[161] Steck-Scott S., Arab L., Craft N.E., Samet J.M. (2004). Plasma and lung macrophage responsiveness to carotenoid supplementation and ozone exposure in humans. Eur. J. Clin. Nutr..

